# Paradoxical Immune Reconstitution Inflammatory Syndrome Presenting as Extrapulmonary Tuberculosis in a Patient With HIV

**DOI:** 10.7759/cureus.100745

**Published:** 2026-01-04

**Authors:** Wendy Shang, Tony F Bruno

**Affiliations:** 1 Department of Biomedical Education, California Health Sciences University College of Osteopathic Medicine, Clovis, USA; 2 Department of Microbiology and Infectious Disease, HIV Program, Doctors With Africa Collegio Universitario Aspiranti Medici Missionari (CUAMM), Padova, ITA

**Keywords:** hiv aids, hiv aids antiretroviral therapy, hiv sub-saharan africa, immune reconstitution inflammatory syndrome (iris), tb-iris

## Abstract

Immune reconstitution inflammatory syndrome (IRIS) is an uncoordinated hyperinflammatory response to latent or de novo active infection in immunocompromised individuals following immune recovery. More specifically, paradoxical IRIS refers to the deterioration of a pre-existing infection, whereas unmasking IRIS can be thought of as the unveiling of a previously undiagnosed infection as immune function reconstitutes. HIV-positive patients with advanced disease who are also coinfected with tuberculosis have a uniquely high risk for this complication once started on highly active antiretroviral therapy (HAART).

We present a case of a 58-year-old HIV and tuberculosis (TB)-positive woman from Northern Tanzania who presented to a rural care and treatment clinic (CTC) after a 17-month lapse from HAART. She had previously undergone curative treatment for tuberculosis three years prior, which was also the time of her initial HIV diagnosis and initiation of HAART. She arrived in the clinic cachectic, unwell, and with complaint of malaise and diarrhea. Blood analysis confirmed advanced HIV disease (AHD), and consequently, she was immediately restarted on HAART. Almost three weeks later, she returned to the clinic febrile, severely fatigued, and with grossly evident cervical lymphadenitis. Given her coinfection (HIV and TB positive) history, depleted CD4+ count, new onset of clinical findings, and temporal relationship of symptom appearance from HAART reinitiation, a diagnosis of paradoxical TB-IRIS presenting as TB lymphadenitis was made.

## Introduction

*Mycobacterium tuberculosis* (TB) infection remains the world’s most deadly infectious disease, with 10 million cases and nearly 1.5 million deaths in 2018 alone [1]. Global rates of HIV-TB coinfection vary widely, ranging from approximately 3% in some regions to as high as 65-72% in others [2,3]. Lower to middle-income countries, particularly those in sub-Saharan Africa, bear the greatest burden of this coinfection [4]. Since TB infection leads to increases in HIV replication and HIV infection contributes to TB progression secondary to immunosuppression, it should come as no surprise that TB burden remains highest in areas of the world that hold the highest HIV prevalence [2]. People living with HIV have a 19 times increased risk of developing TB and make up 17% of all TB deaths worldwide [3]. TB accounts for approximately one in three AIDS-related deaths, making it the leading contributor to mortality in people infected with HIV [1].

The introduction of highly active antiretroviral therapy (HAART) over the past 25 years has substantially reduced mortality in individuals living with HIV worldwide; however, HAART has also introduced new challenges, including immune reconstitution inflammatory syndrome (IRIS), a poorly understood condition whose exact mechanism is not yet fully known [1]. It is best described as a state of dysregulated, hyperinflammatory response against opportunistic infection following the restoration of immune function. This dysregulated immune activation can lead to significant morbidity and, in severe cases, disability or even death [5]. On the other hand, interrupting HAART in a patient with IRIS may expose the patient to the risk of acquiring new opportunistic infections, recurrence of IRIS upon reinitiation of therapy, and possible HIV-drug resistance [5]. Ever since the implementation of HAART, the rate of TB-IRIS seen in HIV-positive patients has significantly increased, and this condition is emerging as an important clinical challenge in resource-limited settings [4]. Some studies suggest TB-IRIS prevalence rates as high as 20-30% in HIV-positive individuals, while the presence of IRIS in similarly immunocompromised HIV-positive individuals is currently unknown [6].

In 2006, the International Network for the Study of HIV associated IRIS (INSHI) established case definitions for the following two different forms of TB-IRIS: paradoxical and unmasking [7]. Paradoxical TB-IRIS refers to clinical or radiological worsening of previously diagnosed TB after HAART initiation despite adequate anti-tubercular therapy (ATT) [5]. This is distinct from a TB paradoxical reaction, which may occur after initiating ATT and is unrelated to immune restoration and therefore also occurs in individuals regardless of HIV status. Paradoxical TB-IRIS is seen in patients with TB infection (active or latent) undergoing HAART treatment, and unmasking TB-IRIS is a complication that takes place in patients with occult or subclinical TB that has not yet been diagnosed [5,8].

Early in the HAART era, IRIS case definitions emphasized laboratory markers such as high viral loads and low CD4 T-cell counts; however, this approach was impractical in low-resource settings, which are the very settings most affected by TB-IRIS, as advanced diagnostic capabilities are often unavailable [7]. To address these limitations, the case definition of paradoxical TB-IRIS is under constant evolution. Significant strides have been made lately using latent class analysis modeling, which helps identify signs and symptoms that are themselves imperfect predictors of disease, but when occurring in clusters are extremely accurate and validated as predictors [4]. In the absence of available diagnostic tests for TB-IRIS, diagnosis relies on incorporating clinical and laboratory data in the context of excluding alternative causes. Some examples of conditions that may have a similar clinical picture to IRIS are drug resistance, non-adherence, drug toxicity, or the possibility of an infection with opportunistic microbes. Our patient was diagnosed with paradoxical TB-IRIS in accordance with case definitions developed by latent modeling analysis and proposed by the International Network on the Study of IRIS [4].

## Case presentation

A 58-year-old HIV-positive female presented to the Care and Treatment Clinic (CTC) at Songambele Hospital in Northern Tanzania after a 17-month lapse from antiretroviral therapy. She had previously been treated successfully for both pulmonary and extrapulmonary tuberculosis three years prior, coinciding with her initial treatment with HAART. During that time, she demonstrated excellent adherence and clinical improvement for approximately 1.5 years. After a 17-month absence from HAART treatment, the patient returned to the clinic reporting malaise, fatigue, and diarrhea, while denying any cough, fever, night sweats, or shortness of breath. Physical examination at that time was grossly normal to all systems. Bloodwork revealed a CD4 T-cell count of 122 cells/μL, indicating advanced HIV disease (AHD), a state of significant immune suppression. HAART was promptly restarted with zidovudine, lamivudine, and nevirapine (AZT/3TC/NVP), the same regimen she had been receiving previously.

**Figure 1:**
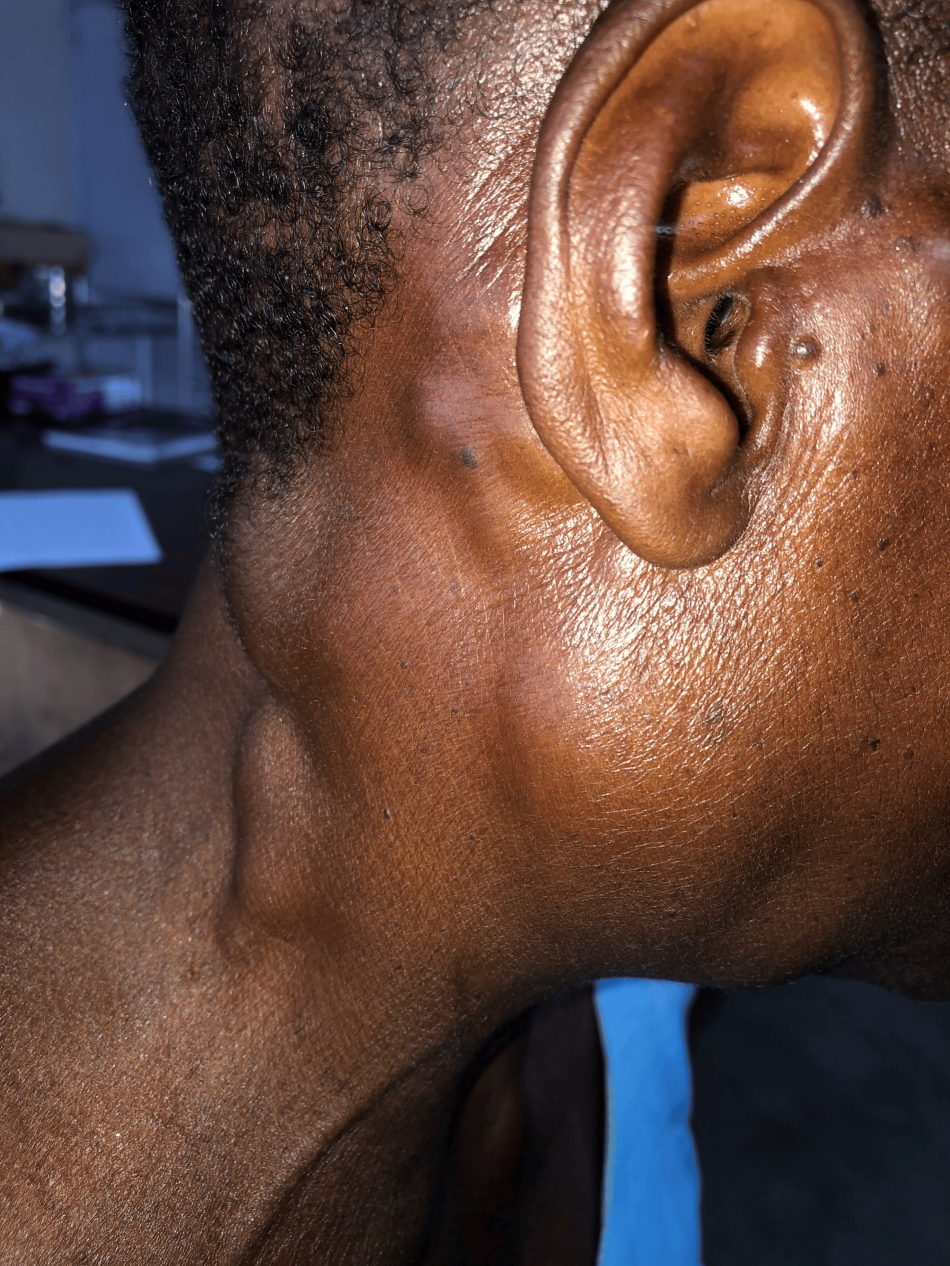
Cervical lymphadenitis involving the superficial cervical, deep cervical, and posterior cervical chains.

Three weeks later, she returned with new complaints of worsening malaise, generalized joint pain, and palpable lumps in her neck. She denied weight loss and night sweats. Her vital signs were consistent with an acute inflammatory reaction or infection (temperature=38.1°C, heart rate=112 beats per minute, respiratory rate=18 breaths per minute, blood pressure=142/90 mmHg, oxygen saturation=93% on room air). The systems-based physical examination was grossly unremarkable, except for the head and neck examination. Grossly evident in the lateral and submandibular areas of the neck were numerous discrete masses and areas of swelling. Palpation revealed multiple painful, warm, mobile, marble-sized (1-2 cm) masses consistent with reactive lymph nodes in the submandibular, parotid, posterior auricular, cervical, and occipital regions (Figure 1).

Given her previous positive history of TB, low CD4 count (and likely concomitant high viral load), as well as onset timing of symptoms in relation to the reinitiation of HAART, a diagnosis of paradoxical TB-IRIS, specifically TB lymphadenitis, was made. Due to our limited diagnostic resources, the patient was transferred to Aga Khan Hospital in Mwanza, where our diagnosis was supported by a biopsy and GeneXpert (Sunnyvale, CA: Cepheid) testing.

## Discussion

This case illustrates paradoxical TB-IRIS in an HIV-positive patient who had been lost to follow-up for 17 months and developed reactive cervical lymphadenitis shortly after restarting HAART. The patient’s HIV clinical course reflects a pattern frequently observed in resource-limited settings, where physical or social barriers can often lead to interruption of HAART treatment for a period of time and, in doing so, create a weakened immune state at elevated risk for triggering IRIS upon reinitiation of therapy.

TB-IRIS typically occurs within days to weeks, and up to a few months, after the initiation or reinitiation of HAART, particularly in individuals with low baseline CD4 counts and high mycobacterial burden [7]. Our patient’s CD4 count of 122 cells/μL, in combination with a corresponding high viral load, placed her at high risk for IRIS. The absence of pulmonary symptoms and the presence of peripheral lymphadenopathy pointed toward extrapulmonary tuberculosis, manifesting as TB lymphadenitis, which is a known presentation of paradoxical TB-IRIS. It is interesting to note that the location of lymphadenitis due to IRIS in our patient is the most common site of extrapulmonary tuberculosis. Although cervical TB or scrofula can present similarly, it is important to understand that the pathophysiologic processes are distinctly different. There is no active infection in our patient; however, there has been development of a hyperimmune response to persistent antigens that have gone awry.

IRIS has a heterogeneous presentation and can be difficult to diagnose. The following conditions can closely mimic IRIS and must be considered or excluded before the diagnosis of IRIS is made. Alternative conditions may include: (1) non-adherence to medication, (2) medication side effects, (3) infection not yet diagnosed, and even (4) malignancy. We were confident that non-adherence was not the cause of this patient’s symptoms, as we conduct compliance monitoring of the patient's medications at every clinic visit for all patients. We reassured ourselves that the proper regimen had been followed. We also believed that the patient’s symptoms were unlikely due to the HAART medication itself, since it is the identical regimen that she was treated with years prior and tolerated well without side effects. While unlikely, malignancy could not be fully ruled out or dismissed at this time, although it is certainly uncommon for most cancers to induce such a severe degree of lymphadenopathy as we observed in this patient in such a short period. In the end, the patient’s lymph nodes were noted to be quite painful, a finding uncommon with malignancy presentation. Taken together, and for the reasons listed above, we felt that the most likely diagnosis was paradoxical IRIS.

Our diagnosis was developed as the degree of suspicion, based on several factors, evolved. These included the presence of a well-documented history of HIV-TB coinfection and initiation of the current HAART regimen while the patient was in a high-risk state for IRIS, namely marked immunosuppression secondary to significant CD4 T-cell depletion. Emerging from such a state of AHD immunosuppression has been linked to increased risk for the development of IRIS [7]. The consideration of each of these elements, as well as the ruling out of other possible etiologies, collectively supported our diagnosis. Access to advanced diagnostic tests, such as bacterial growth capabilities or the presence of diagnostic imaging or histopathology, is uniformly limited in areas like rural Tanzania. Similarly, except for mild cases of IRIS where the mainstay of management is supportive care, the lack of medication availability, such as steroids or immunosuppressants, necessitates prompt referral to tertiary centers for inpatients displaying moderate or advanced cases. The patient’s referral to Aga Khan Hospital was crucial for confirming the diagnosis and initiating appropriate TB therapy alongside continued HAART.

Current case definition of paradoxical TB-IRIS

Understanding the contemporary diagnostic framework for case definitions of paradoxical TB-IRIS remains essential when evaluating patients in these resource-limited settings. There are three major components of the current definition used for paradoxical TB-IRIS (Table 1) [4]. There are antecedent requirements and clinical criteria (major and minor) that must be present in the absence of any other explanation for clinical deterioration. Two antecedent criteria are required for paradoxical TB-IRIS, and both must be present to establish the diagnosis. The first is that there must be a confirmed clinical diagnosis of tuberculosis before the initiation of HAART, and the second is that there must be clear clinical amelioration of the patient's symptoms after starting anti-tubercular therapy (ATT). Clearly, our patient met both antecedent requirements. The clinical criteria in this case definition require that symptoms occur within the first 90 days of starting antiretroviral therapy (HAART) [4,6]. Specific to our patient, severe lymphadenopathy was the major criterion, and fever, fatigue, and general malaise comprised the minor criteria.

**Table 1:** Case definitions for paradoxical TB-IRIS. TB-IRIS: tuberculosis-immune reconstitution inflammatory syndrome; ATT: anti-tubercular therapy; HAART: highly active antiretroviral therapy

Component	Criteria	Requirement
Antecedent requirements	(A) Confirmed clinical diagnosis of tuberculosis before HAART initiation and (B) clear clinical improvement following ATT initiation	Both criteria must be met
Clinical criteria	(A) Development or worsening of TB-related symptoms (pulmonary or extrapulmonary) within 90 days of starting HAART and (B) includes major and minor clinical features - (i) major clinical features: new or enlarging lymph nodes, cold abscesses, or other focal tissue involvement; new or worsening radiologic features of TB; new or enlarging CNS tuberculomas; new or worsening serositis and (ii) minor clinical features: new or worsening constitutional symptoms; new or worsening respiratory symptoms; new or worsening abdominal pain not explained by another cause	At least one major or minor feature
Exclusion of alternatives	(A) Deterioration must not be due to TB drug resistance, ATT non-adherence, HAART or ATT toxicity, or other opportunistic infections or malignancies	Other causes must be excluded

Evidence for the management of TB-IRIS remains limited; however, a meta-analysis review of over 1000 cases of TB-IRIS clearly demonstrates that corticosteroid therapy can significantly reduce associated morbidity [9]. Adjunctive therapies, including prolonged anti-tubercular treatment and careful continuation of HAART, form the cornerstone of management. Most experts would recommend continuing HAART alongside appropriate antimicrobial therapy for reactivated infection, except in severe cases, or during which temporary cessation of HAART may be indicated [8,10]. In addition to corticosteroids, other medications can be considered in specific clinical scenarios. Agents targeting interleukin-6 (IL-6) have emerged as potential options. Though there have been no randomized trials yet to support routine use in TB-IRIS, data suggest that IL-6 plays a pivotal role in the inflammatory cascade, making IL-6 modulators a biologically plausible treatment target [11].

Importantly, this case also highlights a critical public health challenge, the issue of loss to follow-up in HIV treatment. Patients who initially respond well to treatment often stop treatment once they feel better, leading to disease progression and poor outcomes. Structural and social barriers, including stigma, cost, and distance to health facilities, may also contribute to poor long-term HAART adherence. Consistent patient counseling on the importance of lifelong HAART, regular community health worker outreach, and reliable follow-up tracking are vital to improving long-term retention and preventing complications such as IRIS [12].

## Conclusions

TB-IRIS should be strongly considered in HIV patients presenting with new systemic symptoms within weeks of HAART initiation, especially in those with a known history of TB and documentation of significantly reduced CD4 counts. This case supports the continued need for interventions aimed at improving HAART adherence and retention in care. Further, it emphasizes the diagnostic challenges of TB-IRIS in resource-limited areas, the very settings where the prevalence of this phenomenon is highest. Nonetheless, we have demonstrated that the ability to make provisional diagnoses of paradoxical IRIS in settings where only point-of-care testing is available can be accomplished. A diagnosis of IRIS can be established with the mere convergence of confirmed depleted CD4^+^ counts suggestive of an immunocompromised host, the presence of common signs by physical presentation, and the application of critical medical reasoning. In our patient, the importance of a well-taken history and chart review cannot be overstated since this was integral to considering the diagnosis to begin with. Perhaps there is no more profound advice warranted for diagnosing IRIS in similar settings than that given by the father of modern medicine, Sir William Osler, famously accredited with “listen to your patient - he is telling you the diagnosis.” Clinicians should maintain a high index of suspicion when treating patients coinfected with HIV and TB and ensure timely referral to higher-level care facilities when advanced diagnostics and therapies are required.

## References

[1] World Health Organization. (2019 (2025). Global tuberculosis report: executive summary. https://cdn.who.int/media/docs/default-source/documents/tuberculosis/global-tb-report-2019-executive-summary7248223a-1e25-42a6-ada7-a9503c31bb77.pdf.

[2] World Health Organization. (2022 (2025). Global tuberculosis report. http://who.int/teams/global-programme-on-tuberculosis-and-lung-health/tb-reports/global-tuberculosis-report-2022.

[3] Belay M, Bjune G, Abebe F (2015). Prevalence of tuberculosis, HIV, and TB-HIV co-infection among pulmonary tuberculosis suspects in a predominantly pastoralist area, northeast Ethiopia. Glob Health Action.

[4] Stek C, Buyze J, Menten J (2021). Diagnostic accuracy of the INSHI consensus case definition for the diagnosis of paradoxical tuberculosis-IRIS. J Acquir Immune Defic Syndr.

[5] Walker NF, Scriven J, Meintjes G, Wilkinson RJ (2015). Immune reconstitution inflammatory syndrome in HIV-infected patients. HIV AIDS (Auckl).

[6] Thapa S, Shrestha U (2023). Immune reconstitution inflammatory syndrome. StatPearls [Internet].

[7] Meintjes G, Lawn SD, Scano F (2008). Tuberculosis-associated immune reconstitution inflammatory syndrome: case definitions for use in resource-limited settings. Lancet Infect Dis.

[8] Müller M, Wandel S, Colebunders R, Attia S, Furrer H, Egger M (2010). Immune reconstitution inflammatory syndrome in patients starting antiretroviral therapy for HIV infection: a systematic review and meta-analysis. Lancet Infect Dis.

[9] Namale PE, Abdullahi LH, Fine S, Kamkuemah M, Wilkinson RJ, Meintjes G (2015). Paradoxical TB-IRIS in HIV-infected adults: a systematic review and meta-analysis. Future Microbiol.

[10] Vignesh R, Balakrishnan P, Tan HY, Yong YK, Velu V, Larsson M, Shankar EM (2023). Tuberculosis-associated immune reconstitution inflammatory syndrome - an extempore game of misfiring with defense arsenals. Pathogens.

[11] Hamilton F, Schurz H, Yates TA (2025). Altered IL-6 signalling and risk of tuberculosis: a multi-ancestry Mendelian randomisation study. Lancet Microbe.

[12] Quinn CM, Poplin V, Kasibante J, Yuquimpo K, Gakuru J, Cresswell FV, Bahr NC (2020). Tuberculosis IRIS: pathogenesis, presentation, and management across the spectrum of disease. Life (Basel).

